# Is Alcohol Food?

**Published:** 1881-09

**Authors:** 


### IS ALCOHOL FOOD?

WHEN the renowned Doctor Sangrado proclaimed that in certain conditions of the nervous system his patients, when angry, emitted perfumes resembling pine-apple, vanilla, etc., he no doubt expected that its absurdity would cause people to laugh; and when a wag suggested that a man might utilize his mother-in-law by stirring her up and bottling the product, it kept up the laugh, and no doubt gave the doctor a notoriety that "paid." But when a medical man of standing asserts that experiments made on himself prove that alcohol is food he states an absurdity, but an absurdity that is powerful for mischief, since half the world will readily believe it, and the other half won't take the trouble to deny it.

The truth is, that experiments commenced more than six thousand years ago, and continued industriously during these intervening ages, on more than five hundred million human subjects, prove that alcohol in all its forms, when taken into the system, interferes with the processes of digestion and elimination. In plain English, the various functions of the body "get drunk" and fail to carry on healthfully the processes of life. The excretive process becomes torpid, and more or less of the products of combustion—the dead and effete matter—is retained in the system; this adds bulk and weight to the body, and many a hard drinker flatters himself that he is gaining in flesh and in health when he is really but carrying a dead carcass within him. The vulgar term "bloat" when applied to such persons is too near the truth to be considered a joke.

This is only one of the inevitable penalties of drinking. The drunkard, like "Pandora's box," is filled with evils, and at last "Hope" itself leaves the wreck.

It is too late to offer compromise or consolation to this monstrous evil by any legerdemain intended to convert vile grog into beef and mutton and bread.

